# Small RNA sequencing provides insights into molecular mechanism of flower development in *Rhododendron pulchrum* Sweet

**DOI:** 10.1038/s41598-023-44779-z

**Published:** 2023-10-20

**Authors:** Bo Fang, Zhiwei Huang, Yirong Sun, Wanjing Zhang, Jiaojun Yu, Jialiang Zhang, Hongjin Dong, Shuzhen Wang

**Affiliations:** College of Biology and Agricultural Resources, Huanggang Normal University, Huanggang, 438000 Hubei People’s Republic of China

**Keywords:** Molecular biology, Plant sciences

## Abstract

*Rhododendron pulchrum* sweet, a member of the Ericaceae family possessing valuable horticultural properties, is widely distributed in the temperate regions. Though serving as bioindicator of metal pollution, the molecular mechanism regulating flowering in *R. pulchrum* is very limited. Illumina sequencing was performed to identify critical miRNAs in the synthesis of flavonoids at different developmental stages. Totally, 722 miRNAs belonging to 104 families were screened, and 84 novel mature miRNA sequences were predicted. The miR166, miR156, and miR167-1 families were dominant. In particular, 126 miRNAs were significantly differentially expressed among four different flowering stages. Totally, 593 genes were differentially regulated by miRNAs during the flower development process, which were mostly involved in “metabolic pathways”, “plant hormone signal transduction”, and “mitosis and regulation of biosynthetic processes”. In pigment biosynthesis and signal transduction processes, gra-miR750 significantly regulated the expression of flavonoid 3’,5’-hydroxylase; aof-miR171a, aof-miR171b, aof-miR171c, cas-miR171a-3p, and cas-miR171c-3p could regulate the expression of DELLA protein; aof-miR390, aof-miR396b, ath-miR3932b-5p, cas-miR171a-3p, aof-miR171a, and aof-miR171b regulated BAK1 expression. This research showed great potentials for genetic improvement of flower color traits for *R. pulchrum* and other *Rhododendron* species.

## Introduction

*Rhododendron* genus contains approximately 1000 evergreen and deciduous species, as well as thousands of commercial hybrids, which is widely distributed in Europe, Asia, and North America^1,2^. As a typical large vascular plant genus within the Ericaceae family, *Rhododendron* possess valuable medicinal and horticultural properties^3,4^. Besides bioindicator of metal pollution (Pb, Zn, and Cd), the evergreen *R. pulchrum* is also famous due to the beautiful vegetative forms, bright-colored flowers, and long flowering period ranging from late March to early May^5^. Therefore, *R. pulchrum* is widely served as roadside trees in urban and rural areas^5^.

During the past three decades, genetic analyses and transcriptomics studies have identified several hundred genes involved in flower development, and the majority encode transcription factors and transcriptional co-regulators, proteins involved in epigenetic control of gene expression, and microRNAs (miRNAs)^6,7^. In particular, transcription factor-encoding genes AINTEGUMENTA (ANT), auxin efflux carrier Pin-Formed 1 (PIN1), Aintegumenta-like 6 (AIL6), regulatory gene LEAFY (LFY), MADS-domain transcription factors Agamous-like 24 (AGL24), Short Vegetative Phase (SVP), and shoot identity gene Terminal Flower1 (TFL1) were all deeply studied^8,9^. Based on major advances in technology, genome-wide studies of transcription factor-binding sites, proteomic analyses, and imaging techniques have all been adopted to clarify molecular mechanisms underlining flowering^10^.

MiRNAs (approximately 22 nt), a class of non-coding single strand molecules negatively regulating gene expressions both at transcriptional and posttranscriptional levels, play critical roles in biological processes, including embryogenesis and organ development^11–14^, and metabolism^15–18^. In particular, functional mature miRNAs could be incorporated into Argonaute 1 (AGO1)-containing RNA-induced silencing complex (RISC), and causing degradation or translational repression in sequence-specific manner^19,20^. The miRNAs often regulate corresponding mRNA targets through guiding cleavages between 10 and 11th nucleotides in complementary region^21^.

Though molecular methods have been utilized to study the growth, development, and metabolism regulation, the basic molecular research of *R. pulchrum* is still limited, especially for the molecular mechanisms underlying anthocyanin synthesis. In order to explore the potential roles of miRNAs during flowering process, miRNA expression profiles of *R. pulchrum* flowers at four different developmental stages were investigated with high throughput small RNA sequencing technology. Differentially expressed miRNAs among different flower developmental stages and corresponding targets were also clarified. This research will be benefit for genetic improvement of *R. pulchrum* and other *Rhododendron* species.

## Results

### Sequence analysis of small RNA in *R*. *pulchrum* flowers

**Figure 1:**
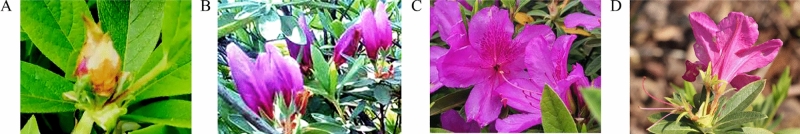
Flower tissues of four different stages: (**A**–**D**) representing stage I (floral bud stage), stage II (early flowering stage), stage III (full-flowering stage), and stage IV (flower withering stage), respectively.

*R. pulchrum* flowers at four different developmental stages were separately collected, including stage I (floral bud stage, late-March, dormant flower buds, floral organs had been well formed containing style, petal primordium, stamen primordia, sepal primordia, pistil primordia, and ovary), stage II (early flowering stage, early-April, 1–2 days before full bloom), stage III (full-flowering stage, middle-April, completely open petals, observable pistils and stamens), and stage IV (flower withering stage, late-April, petals began to fall) (Fig. 1). Twelve small RNA libraries (three libraries for each developmental stage) were constructed and sequenced. Totally, 28,256,644–33,991,292, 32,259,303–39,111,962, 29,838,331–36,968,844, and 29,218,896–41,838,094 raw reads were generated; while 25,977,837–31,816,069, 28,096,248–32,886,303, 22,332,972–27,916,820, and 23,035,469–33,163,852 clean reads (≧18nt) were obtained after removing low quality reads and adaptors for stage I, stage II, stage III, and stage IV respectively (Table 1). The Q20 values were all above 99.68%. GC contents varied from 51.52% (stage I) to 55.04% (stage III). In particular, similar length distributions of small RNA were observed for four set samples of different developmental stages. Among 18–40 nt small RNAs, the majority ranged from 21 to 25 nt in length, and the percentages ranged within 48.24–84.15%. In particular, the most abundant sRNAs were 24nt, and the percentages of 24 nt sRNAs were 59.46%, 37.06%, 20.03%, and 19.12% for stage I, stage II, stage III, and stage IV respectively. However, the second abundant sRNAs were 25 nt in stage I samples, but 21 nt for the rest samples. The third abundant sRNAs were 22 nt in samples at stage II, stage III, and stage IV; while sRNAs with length of 21 nt was the third abundant type for stage I samples (Fig. S1).

**Table 1:** Small RNA categorization in *R. pulchrum* flowers at different developmental stages.

RNA type	Stage I	Stage II	Stage III	Stage IV
Raw reads	28,256,644–33,991,292	32,259,303–39,111,962	29,838,331–36,968,844	29,218,896–41,838,094
Clean reads	25,977,837–31,816,069	28,096,248–32,886,303	22,332,972–27,916,820	23,035,469–33,163,852
Q20	99.69–99.8%	99.72–99.79%	99.68–99.71%	99.7–99.71%
GC%	51.52–51.89%	52.71–52.75%	54.88–55.04%	54.06–54.15%
Mapped percentage	75.34%	78.24%	90.39%	87.04%
Annotation reads	19,940,384–24,302,325	22,623,935–26,572,199	21,446,057–26,735,509	21,089,468–30,160,793
Annotation percentage	75.3–75.38%	78.2–78.29%	90.25–90.49%	87.04–87.19%
rRNA	209,874–239,374	328,326–355,140	275,098–335,100	370,317–459,196
tRNA	27,892–31,441	33,640–36,550	20,185–23,230	34,532–42,451
snRNA	12,830–14,212	13,143–14,526	7218–8099	11,913–13,908
snoRNA	14,290–15,991	12,979–14,493	7499–8312	10,740–12,315
Known_miRNA	1500–1622	1678–1843	1231–1435	1.454–1533

**Figure 2:**
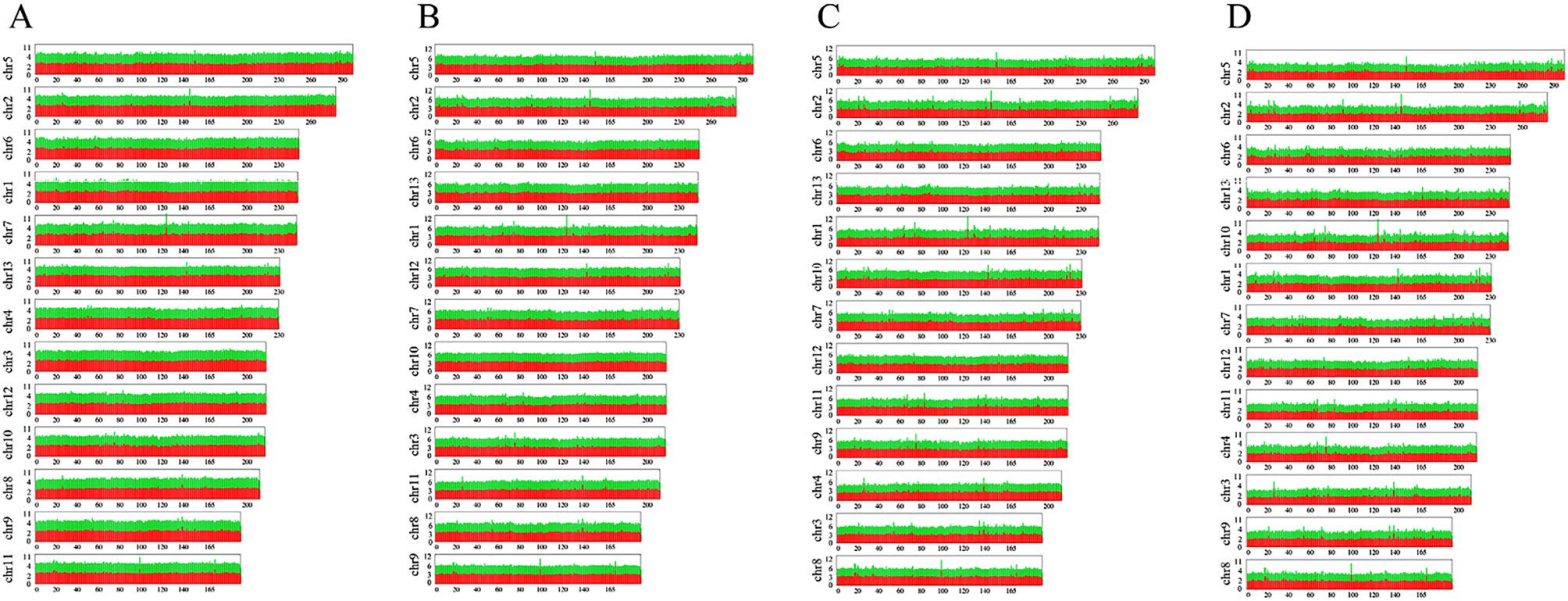
The genome distribution of sRNAs in four flower samples at stage I (**A**), stage II (**B**), stage III (**C**), and stage IV (**D**), respectively. The red parts in Y-axis represents log10 count, and the green part represents log10 category. “Chr” was short for chromosome.

Unique reads were obtained through removing redundant sequence. In total, 75.34%, 78.24%, 90.39%, and 87.04% clean reads could be mapped to the reference genome (*R. simsii* genome) for stage I, stage II, stage III, and stage IV, respectively. Particularly, the most abundant sRNA were mapped to chromosome 5 (9.54–19.2%), chromosome 2 (5.56%-6.71%), and chromosome 6 (4.44–5.12%) of *R. simsii* genome (Fig. 2). After annotation, 19,940,384–24,302,325, 22,623,935–26,572,199, 21,446,057- 26,735,509, and 21,089,468–30,160,793 small RNA tags were obtained for stage I-stage IV samples of *R. pulchrum* flowers, which could be further classified into miRNAs, tRNAs, rRNAs, snRNA, and snoRNA.

### Clarification of miRNAs based on deep sequencing

Totally, 104 miRNA families were obtained, among which miR166 family was the largest represented family with 24 members (Tables S1, S2). Moreover, miR156 (17 members), miR167-1 (17 members), miR159 (16 members), and miR171_1 (16 members) were also the dominant families. Based on alignment results, homologous sequences of these identified miRNA were mainly in *Arabidopsis lyrata*, *Malus domestica*, *Populus trichocarpa*, *Glycine max*, and *Oryza sativa*. The first position of known miRNAs had obvious uracil (U) base preference (Fig. S2A). Besides bases at the first position, the 3’-end bases also possessed U base preference. Moreover, the 8th and 9th bases had slight G base preference, while C base preference existed at 23th position (Fig. S2B).

**Figure 3:**
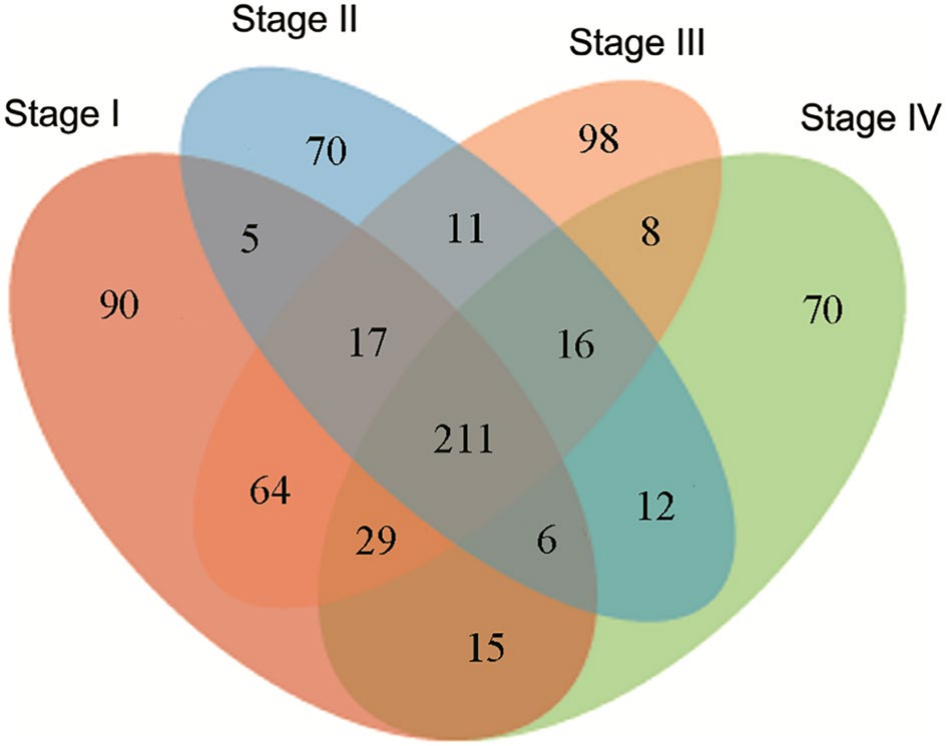
Venn diagram of identified miRNA in four stages.

In related to the 722 known miRNAs, 437, 348, 454, 367 miRNAs were present in stage I, stage II, stage III, and stage IV samples, respectively. Totally, 211 miRNAs regulated expression of corresponding genes across all four developmental stages of *R*. *pulchrum* flowers (Fig. 3 and Table S3). Particularly, 90, 70, 98, and 70 miRNA were unique to stage I, stage II, stage III, and stage IV, respectively. Furthermore, 5, 11, 8, 15, 64, and 12 miRNAs were expressed in “stage I” vs “stage II”, “stage II” vs “stage III”, “stage III” vs “stage IV”, “stage I” vs “stage IV”, “stage I” vs “stage III”, and “stage II” vs “stage IV”, respectively (Fig. 3 and Table S3). Moreover, there were 68 miRNAs were commonly expressed in samples at three developmental stages.

The miRNA sequences, which met the threshold of miRDeep2 analysis but possessing no known homologous miRNA gene families in miRBase, were used to predict novel miRNAs (total score > 0). A total of 84 novel mature miRNA sequences were identified in *R. pulchrum* flowers (Table S4). Totally, four length types of miRNAs were predicted, including 21 nt (55, 65.476%), 22nt (24, 28.571%), 20 nt (4, 4.762%), and 24 nt (1, 1.190%) (Fig. S3A). The first nucleotide showed G and U bias for 20 nt and 21 nt miRNAs, respectively. For these 24 nt miRNAs, the first nucleotide was all U base. Among these 84 novel miRNAs, the 1th, 2th, 7th, and 12th position all had U base preference (Fig. S3B). Bases at 8th and 23th position had obvious C base preference. Moreover, the 24th bases in novel miRNAs were all G base.

### Identification of differentially expressed miRNAs

**Figure 4:**
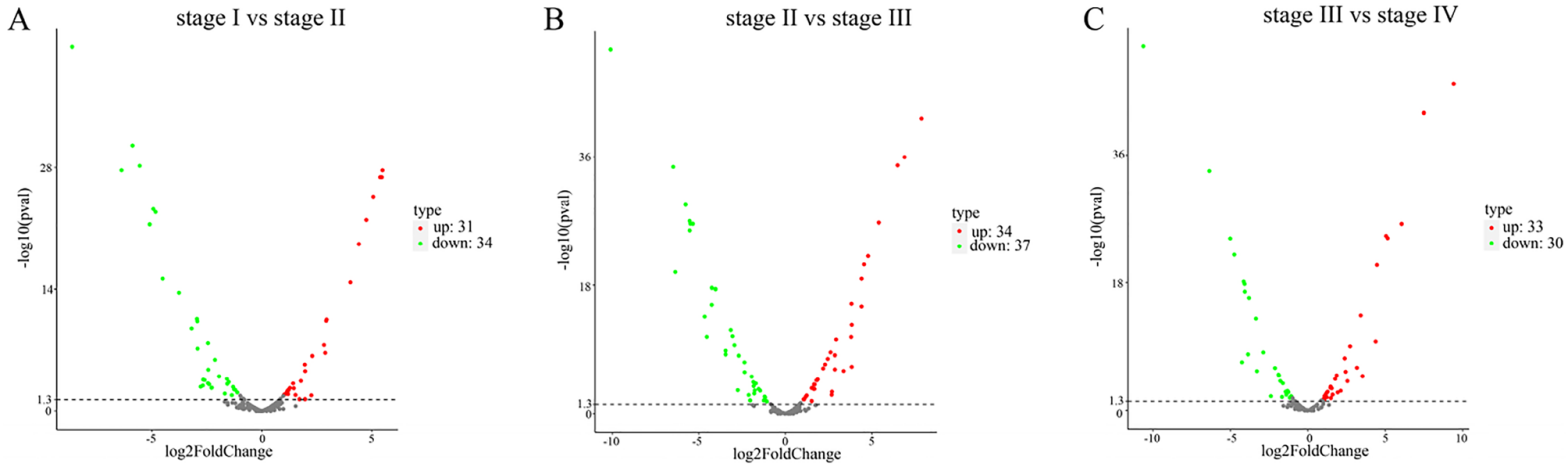
Volcano plots of miRNA data between stage I and stage II (**A**), stage II and stage III (**B**), as well as stage III and stage IV (**C**).

Based on fold change (≥ 1 or ≤ − 1) and P-value (< 0.05) criteria, a total of 126 miRNAs were significantly differently expressed. Compared with samples at stage I, 31 miRNAs were up-regulated and 34 miRNAs were down-regulated in samples at stage II (Fig. 4A and Table S5). Compared with samples at stage II, 34 miRNAs were up-regulated and 37 miRNAs were down-regulated in samples at stage III (Fig. 4B and Table S5). Compared with samples at stage III, numbers of the up-regulated and down-regulated miRNAs in samples at stage IV were 33 and 30, respectively (Fig. 4C and Table S5).

**Figure 5:**
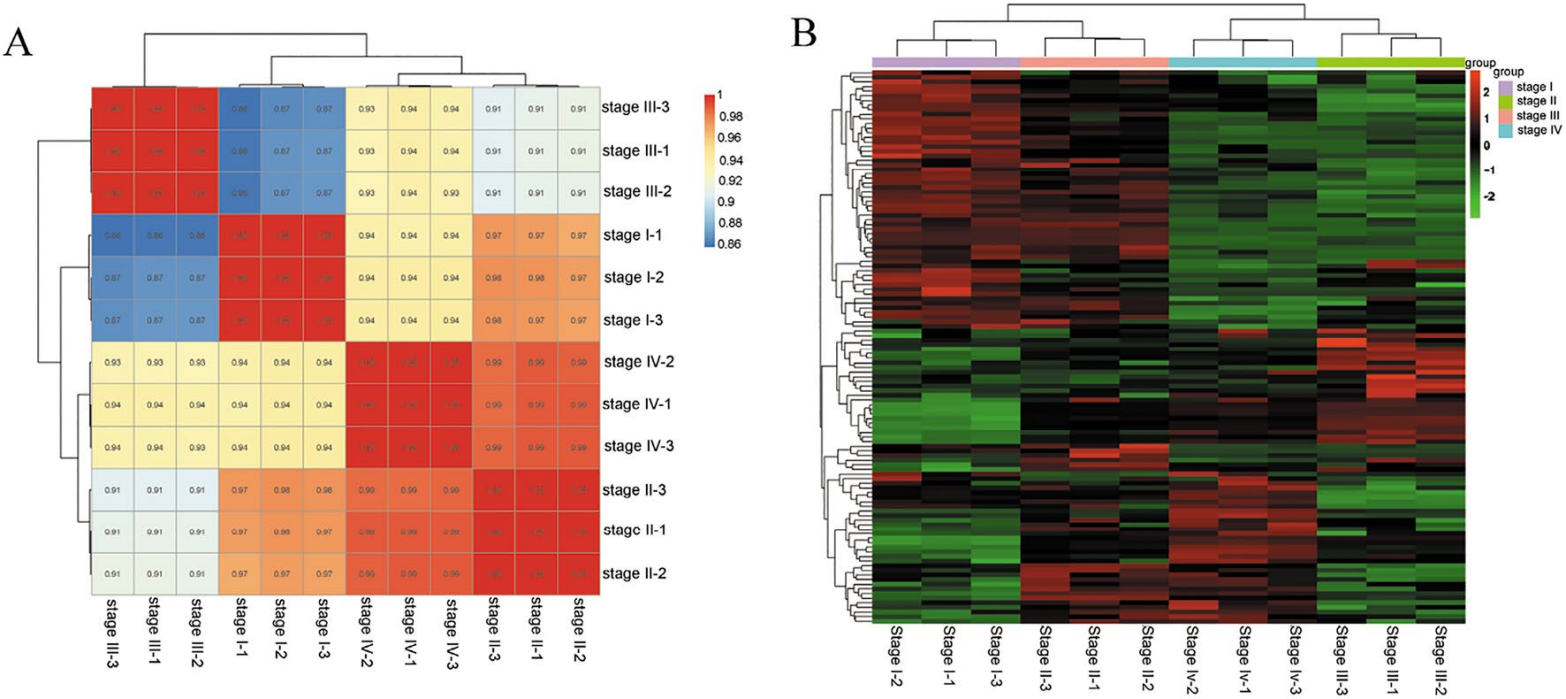
Distance analysis of *R. pulchrum* flower tissues collected from four developmental stages by calculating Pearson’s correlation coefficient: (**A**) sample correlation matrix (*p* < 0.001, *FDR* < 0.001); (**B**) gene cluster analysis (*p* < 0.001, *FDR* < 0.001).

Statistical analysis was carried out through calculating Pearson's correlation coefficient (r) value based on DEGs, and these biological replicates in each group showed high correlation (Fig. 5). Furthermore, miRNA profiles of flower tissues at stage II and stage IV were clustered first, and then grouped with that of flowers sampled at stage I. However, the miRNA profiles of flowers at full-flowering stage (Stage III) showed less association with the other three flower samples (Fig. 5A). Furthermore, gene cluster showed that these DEGs could be mainly clustered into two clades: clades 1 containing miRNA profiles of “Stage I” and “Stage II”, clade 2 consisting of miRNA profiles of “Stage III” and “Stage IV” (Fig. 5B).

### Target prediction and GO enrichment analysis

**Figure 6:**
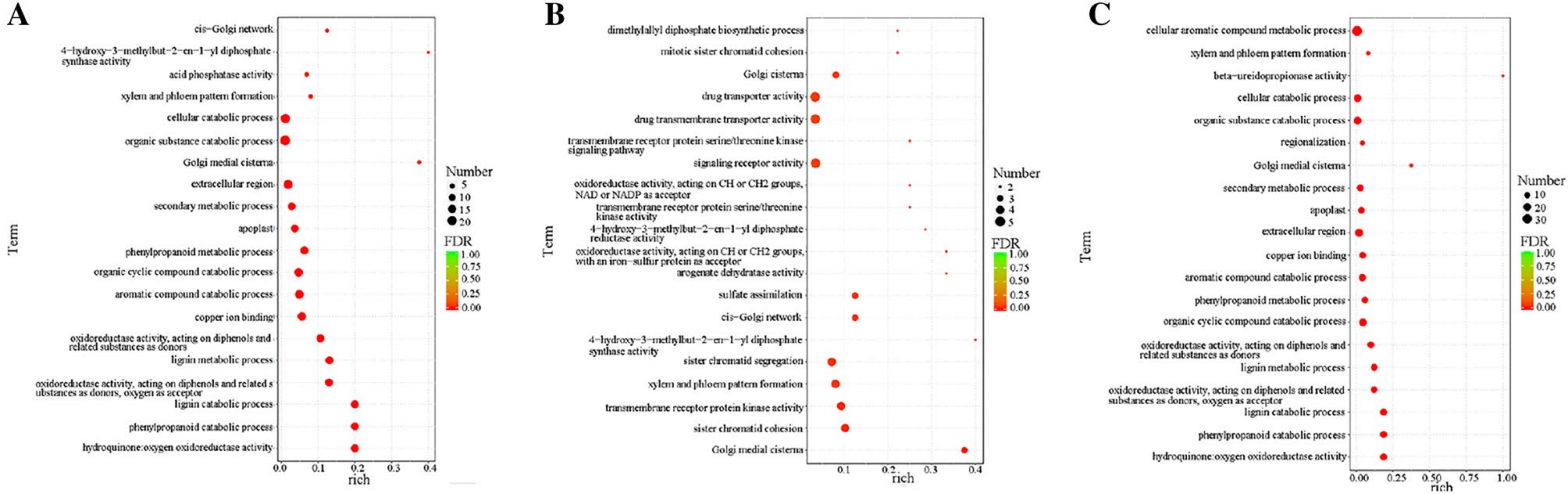
GO annotations of potential miRNA targets during *R. pulchrum* flower development from stage I to stage II (**A**), stage II to stage III (**B**), and stage III to stage IV (**C**).

With transcriptome data of *R. pulchrum* flower obtained in our previous study serving as target database, 1170 miRNA target site (593 target genes) were obtained. Among these miRNA-target pairs, 84.57% of miRNAs potentially targeted mutiple unigenes, ranging from 2 to 157. During *R. pulchrum* flower development from stage I to stage II, a total of 1081 potential miRNA targets were categorized into molecular functions (227), cellular components (109), and biological processes (745) according to GO-based enrichment analysis (Table S6). “organic substance catabolic process” (GO:1901575), “cellular catabolic process” (GO:0044248), and “extracellular region” (GO:0005576) were the main enriched terms (Fig. 6A). Among GO terms associated with biologic process, the target genes were mainly involved in “monosaccharide transporter” and “xenobiotic detoxification” pathways (Fig. S4A). In the cellular component category, target genes were tightly associated with “nucleus and extracellular space” (Fig. S4B). In molecular function, most target genes were related to “isomerase activity”, “electron transfer activity”, and “acid phosphatase activity” (Fig. S4C).

From stage II to stage III, a total of 1328 potential miRNA targets were categorized into molecular functions (258), cellular components (170), and biological processes (900) (Table S6). In particular, “drug transporter activity” (GO:0090484), “drug transmembrane transporter activity” (GO:0015238), and “signaling receptor activity” (GO:0038023) were the most enriched terms (Fig. 6B). Among GO terms related to biologic process, the majority target genes were involved in “histone lysine methylation”, “mRNA splicing”, and “transcription initiation” (Fig. S5A). In cellular component category, target genes were mainly associated with “small-subunit processome assembly”, “1,3-β-d-glucan synthetase complex”, and “nucleolus” (Fig. S5B). For molecular function category, target genes were mainly related to “ADP binding”, “NAD^+^ binding”, and “clathrin binding” (Fig. S5C).

From stage III to stage IV, a total of 951 potential miRNA targets were categorized into molecular functions (169), cellular components (87), and biological processes (695) (Table S6). Particularly, “cellular aromatic compound metabolic process” (GO:0006725), “cellular catabolic process” (GO:0044248), and “organic substance catabolic process” (GO:1901575) were the abundant enriched terms (Fig. 6C). For GO terms associated with biologic process, the target genes were mainly involved in “histone lysine methylation”, “transcription initiation”, and “mRNA splicing” (Fig. S6A). Among cellular component category, target genes were tightly associated with “small subunit processome”, “nucleus”, and “apoplast” (Fig. S6B). In molecular function category, most target genes were related to “NAD + binding”, “copper ion binding”, and “4 iron,4 sulfur cluster binding” (Fig. S6C).

### KEGG pathway analysis of potential targets of known miRNAs

**Figure 7:**
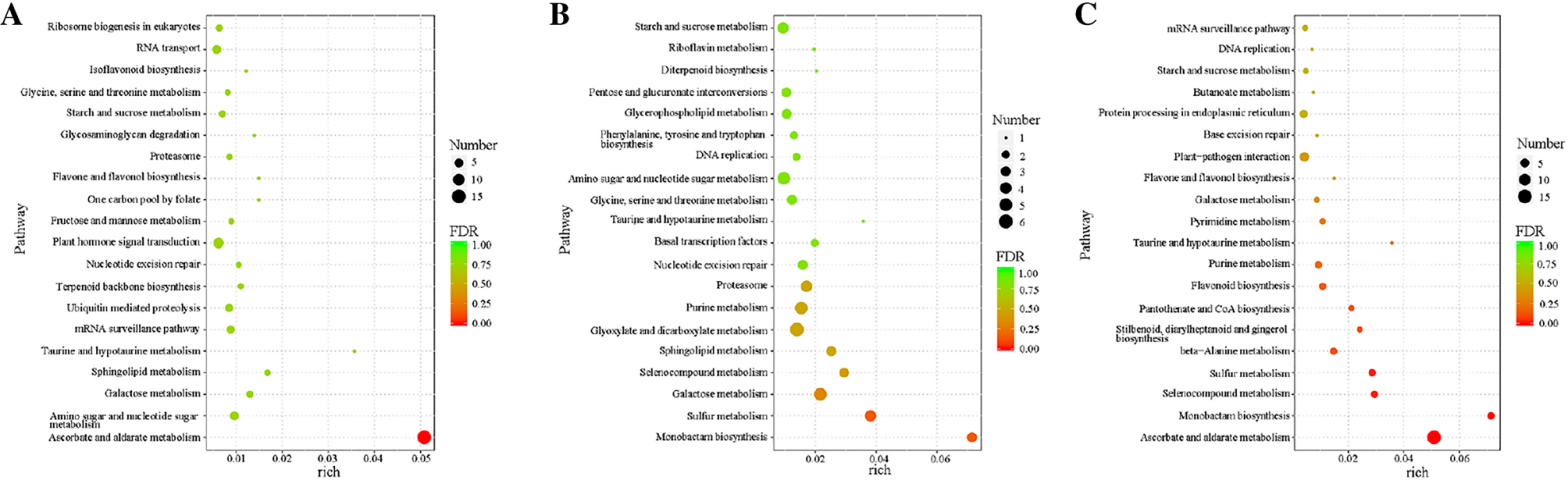
KEGG pathway analysis of potential miRNA targets during *R. pulchrum* flower development from stage I to stage II (**A**), stage II to stage III (**B**), and stage III to stage IV (**C**).

**Figure 8:**
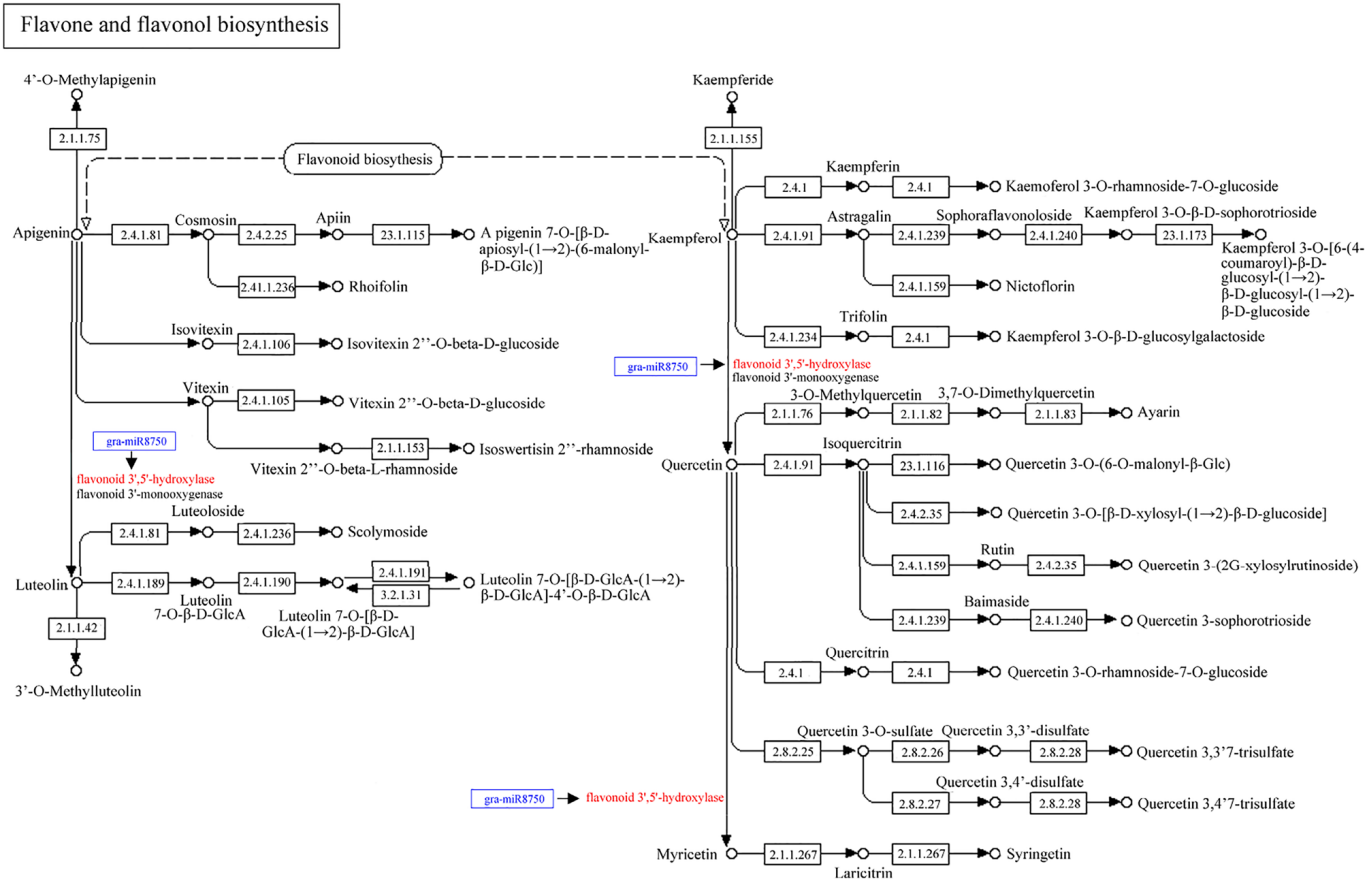
Metabolic regulation of miRNA in the process of flavone and flavonol biosynthesis.

During *R. pulchrum* flower development process from stage I to stage II, 52 pathways have been identified, mainly including “Plant hormone signal transduction” (ko04075), “Ubiquitin mediated proteolysis” (ko04120), “mRNA surveillance pathway” (ko03015), and “Amino sugar and nucleotide sugar metabolism” (ko00520) (Fig. 7A and Table S7). For the “Flavone and flavonol biosynthesis” pathway (ko00944), gra-miR8750 significantly regulated the expression of flavonoid 3’,5’-hydroxylase, which was involved in the biosynthesis of secondary metabolites including 3’-O-Methylluteolin, quercetin, and myricetin (Fig. 8).

**Figure 9:**
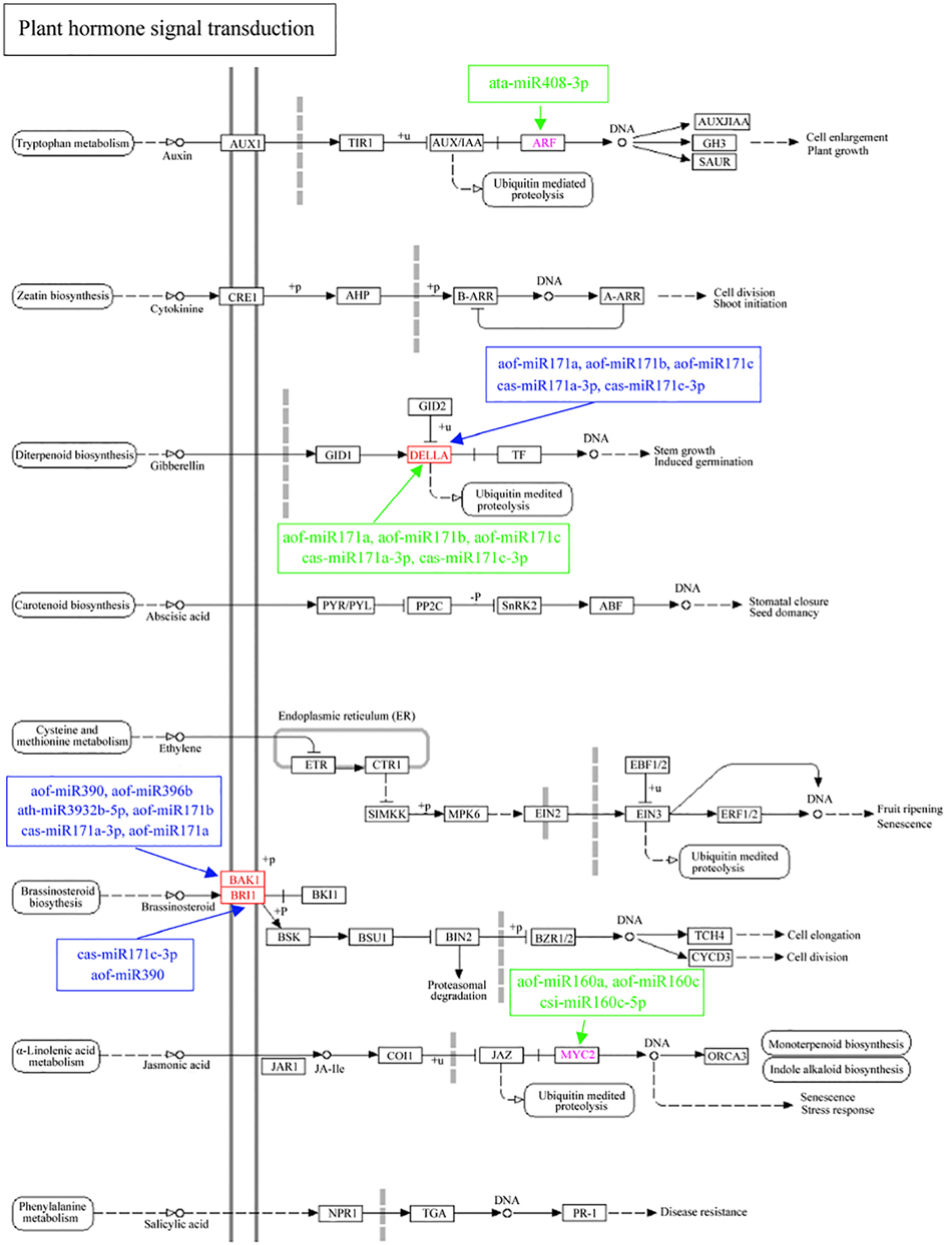
Metabolic regulation of miRNA in the process of plant hormone signal transduction.

During *R. pulchrum* flower development process from stage II to stage III, 65 pathways have been identified, and the most abundant were “Amino sugar and nucleotide sugar metabolism” (ko00520), “Galactose metabolism” (ko00052), “Glyoxylate and dicarboxylate metabolism” (ko00630), and “Plant hormone signal transduction” pathway (ko04075) (Fig. 7B and Table S7). In the “Plant hormone signal transduction” pathway, a list of miRNAs showed great potentials for the signal transduction. During the environmental information processing, aof-miR171a, aof-miR171b, aof-miR171c, cas-miR171a-3p, and cas-miR171c-3p could regulate the expression of DELLA protein, which further affecting the diterpenoid biosynthesis (Fig. 9). Moreover, aof-miR390, aof-miR396b, ath-miR3932b-5p, cas-miR171a-3p, aof-miR171a, and aof-miR171b regulated brassinosteroid biosythesis through changing the expression level of BAK1 protein; while cas-miR171c-3p and aof-miR390 could alter the expression of BR11 protein, a leucine-rich repeat receptor involved in the brassinosteroid (BR) perception (Fig. 9).

During *R. pulchrum* flower development process from stage III to stage IV, 34 pathways were well screened out, mainly involving “Ascorbate and aldarate metabolism” (ko00053), “Plant-pathogen interaction” (ko04626), “Plant hormone signal transduction” (ko04075), and “Protein processing in endoplasmic reticulum” (ko04141) (Fig. 7C and Table S7). For the “Plant hormone signal transduction” (ko04075), auxin response factor (ARF), DELLLA, and MYC2 were significantly regulated by miRNAs (Fig. 9). In particular, the ata-miR408-3p could affect the expression of ARF in tryptophan metabolism. Moreover, these miRNAs consisting of aof-miR171a, aof-miR171b, aof-miR171c, cas-miR171a-3p, and cas-miR171c-3p, all accounted for the diterpenoid biosynthesis through affecting the expression of DELLA protein. Furthermore, aof-miR160a, aof-miR160c, and csi-miR160c-5p regulated the expression of transcription factor MYC2, which was involved in α-Linolenic acid metabolism.

### Validation of differentially expressed miRNAs by real-time qPCR assay

**Figure 10:**
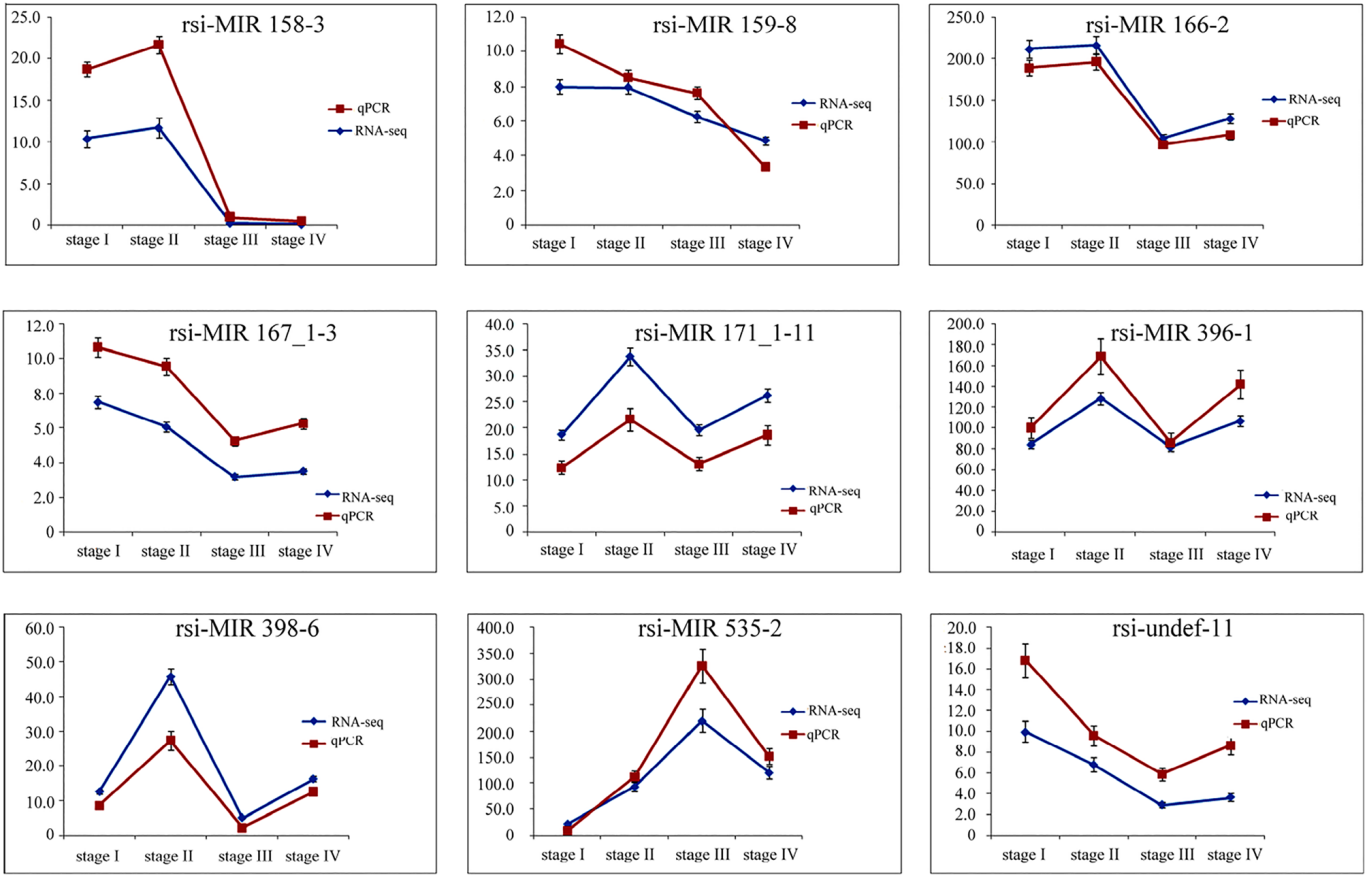
Correlation analyses of nine miRNAs between Illumina sequencing and qRT-PCR data.

To verify the miRNA sequencing data, nine genes exerting diverse expression profiles at four developmental stages were randomly selected for qRT-PCR validation, including rsi-MIR 158-3, rsi-MIR 159-8, rsi-MIR 166-2, rsi-MIR 167_1-3, rsi-MIR 171_1-11, rsi-MIR 396-1, rsi-MIR 398-6, rsi-MIR 535-2, and rsi-undef-1. The qPCR analysis showed that up-regulated and down-regulated results of miRNAs were similar with deep sequencing results (Fig. 10). Good correlation (*R*^2^ = 0.8919) was obtained between sequencing data and qRT-PCR results, further confirmed the high reliability of miRNA sequencing data. Expression pattern of corresponding target genes was also consistent with prediction, which was verified by qRT-PCR analysis (data not shown). The amount of rsi-MIR 159-8, rsi-MIR 167_1-3, and rsi-undef-11 transcript decreased gradually during flowering process, but slightly increased at the withering stage. Moreover, the expression levels of rsi-MIR158-3, rsi-MIR166-2, rsi-MIR171_1-11, rsi-MIR396-1, and rsi-MIR398-6 were all highest at early flowering stage, while transcript of rsi-MIR535-2 was peaked at full-flowering stage.

## Discussion

The miRNAs have been evidenced as key regulators in gene expression, developmental processes, and even stress tolerance^22^. Recently, the bioinformatics, as well as sequencing approaches and technologies have facilitated the rapid and accurate miRNA detection and analysis at single-base pair resolution^23^. In related to the Ericaceae family with great ornamental properties, understanding flowering mechanism will benefit corresponding genetic improvement of flowers traits. However, there is currently limited miRNA information on regulating mechanism underlying flowering of *R. pulchrum*, which impedes further genetic improvement.

In this study, 722 conserved miRNAs representing 104 miRNA families associated with different flower developmental processes were identified in *R. pulchrum* at four different stages. According to Choudhary et al.^24^, the non-coding miRNA might be crucial entity in remodeling genetic architecture of *R. pulchrum*, and the fluctuations in miRNA expression could be induced by developmental factors. The number of miRNAs regulating flower development of *R. pulchrum* (722 known miRNAs and 84 novel miRNAs) is higher than that of Korla fragrant pear at anthesis (73 miRNAs)^25^, that of *Carya cathayensis* genome (51 conserved miRNAs belong to 16 families and 195 novel miRNAs)^26^, as well as that of ornamental *Paeonia rockii* and *Paeonia ostii* petals (22 conserved miRNAs and 27 novel miRNAs)^27^.

Totally, 126 miRNAs were significantly differently expressed, inferring that these miRNAs were inducible and responded to certain developmental stage. The variable miRNA expression among tissues of different developmental stages can be associated with the process of growth and development of *R. pulchrum*. Moreover, exogenous environmental cues also mediate phase transition through networking of flowering pathways as well as corresponding components. In particular, miR166, miR156, miR159, and miR171_1 families were dominant for *R. pulchrum* flower development, which was partly similar with that of maize^28^, *Camellia oleifera*^29^, and *Vigna mung*o^30^. The miR166/165 group and corresponding target genes regulate shoot apical meristem and floral development through WUSCHEL-CLAVATA) pathway^31^. In *Paeonia rockii* petals, variegation formation was regulated by miR168, miR396a, miR159c, and novel_miR_05, which might target MYB transcription factors, CHS, and ABC transporter^27^.

During process of *R. pulchrum* flower development, 1170 miRNA target site (593 target genes) have been screened, much less than that of *R. arboretum* leaves (27,139 predicted targets)^24^. Similarly, these predicted target genes were all involved in metabolism, reproduction, and response to abiotic stimuli. In particular, amino sugar, nucleotide sugar, galactose, glyoxylate, dicarboxylate, ascorbate, and aldarate were all involved in the metabolism processes. Moreover, a negative correlation was observed between the expression of miRNAs and corresponding targets. It further highlighted the critical role of miRNA-target pairs in perceiving environmental variability and monitoring flowering growth. The phylogenetic clustering further supported the lineage-specific evolution and function of putative miRNA sequence in *R. pulchrum*.

Flavonoids are ubiquitous plant secondary metabolites, and their hydroxylation pattern could greatly affect color, stability, and even antioxidant capacity. In particular, hydroxylation pattern of the B-ring of flavonoids is mainly determined by flavonoid 3′-hydroxylase (F3′H) and flavonoid 3′,5′-hydroxylase (F3′,5′H)^32^. As cytochrome P450-dependent monooxygenases, flavonoid 3′,5′-hydroxylase could hydroxylate the B-ring of flavonoids at both 3′- and 5′-position, respectively^33^. In particular, the F3′5′H activity facilitates the synthesis of delphinidin-based anthocyanins, providing the basis for lilac to blue flower colours. During *R. pulchrum* flower development, gra-miR8750 might exert some effects towards the expression of flavonoid 3′,5′-hydroxylase, which further greatly affect flower color.

Gibberellic acid controls several aspects of plant development and growth through GA-GID-DELLA signaling module^34^. With regard to the chlorophyll biosynthetic pathway, DELLA stabilization could lead to increased accumulation of Pchlide and PORs in etiolated seedlings^35^. The miR171 family can destabilize mRNAs encoding SCARECROW-Like transcription factors (SCL6/SCL6-IV, SCL22/SCL6-III and SCL27/SCL6-II), which could cause developmental defects in leaves and flowers^36^. However, SCARECROW-like transcription factors are important for development of roots, where no miR171 was produced^36^. During flower developmental process in *R. pulchrum*, miR171 might also significantly affected the expression of DELLA and BAK proteins, which also deeply validated the importance of miR171 for plant development.

This study contributes a complete profile of miRNAs in *R. pulchrum*. This documentation of genome-wide profiling of miRNAs, their targets, and expression will enhance the understanding of developmental strategies in *Rhododendron* species. Unraveling the complex developmental mechanisms of *R. pulchrum* will benefit the genetic improvement of flower traits in *Rhododendron* species.

## Materials and methods

### Materials

*R. pulchrum* plants used in this research were over 10 years, which were cultivated in Huanggang Botanical garden (114.535° E, 30.278° N, 25 m). We have the permission to collect samples. The samples of *R. pulchrum* flowers at four different developmental stages were separately collected, including stage I (floral bud stage, late-March, dormant flower buds, floral organs had been well formed containing style, petal primordium, stamen primordia, sepal primordia, pistil primordia, and ovary), stage II (early flowering stage, early-April, 1–2 days before full bloom), stage III (full-flowering stage, middle-April, completely open petals, observable pistils and stamens), and stage IV (flower withering stage, late-April, petals began to fall). Flowers collected from three individual plants were pooled as one sample. For each stage, three biological replicates were collected. After being frozen in liquid nitrogen immediately, these samples were stored at − 80 °C until further extraction. The Study complies with local and national regulations and guidelines.

### Small RNA library construction and sequencing

Total RNAs were isolated using TRlzol Reagent (Invitrogen) according to the manufacturer’s instructions. The purity and concentration of RNAs were checked with a Nanodrop 2000 system (Thermo, MA, USA) and a Qubit® RNA Assay Kit in Qubit® 2.0 Fluorometer (Life Technologies, CA, USA), respectively. Moreover, RNA integrity was verified with the RNA Nano 6000 Assay Kit and an Agilent Bioanalyzer 2100 system (Agilent Technologies, CA, USA). Small RNA libraries were constructed with TruSeq Small RNASample prep Kit. After PCR amplification, the enriched libraries were added with sequencing adaptors. Then, the libraries were purified, and checked with Aglient High Sensitivitty DNA Kit on the Agilent Bioanalyzer 2100 system. Quantitative analysis of the libraries were performed with Quant-iT PicoGreen dsDNA Assay Kit. Sequences were carried out on HiSeq 2500 PE125 platform (single-end).

### Identification of miRNAs through deep sequencing

Raw reads (fastq format) were processed through both custom Perl and Python scripts to obtain clean reads. In particular, certain range of lengths were chosen for downstream analysis. Small RNA tags were mapped to the reference genome of *R. simsii* (https://www.ncbi.nlm.nih.gov/genome/94195). The mapped small RNA tags were further aligned to Repeat Masker, Rfam database, and Nr (NCBI nonredundant protein sequences) to remove tags originating from repeat sequences, protein-coding genes, tRNA, rRNA, snRNA, and snoRNA. Afterwards, the remaining tags were aligned with known miRNAs from other plant species in miRBase21.0 and modified by mirdeep2 software^37,38^. Based on hairpin structure of miRNA precursor, novel miRNA were predicted with miREvo and mirdeep2 softwares through exploring secondary structure, dicer cleavage site, as well as minimum free energy of small RNA tags unannotated in the former steps^38,39^. Particularly, the miFam.dat (http://www.mirbase.org/ftp.shtml) was used to screen families of known miRNAs^29^. The miRNA target genes were predicted with psRNATarget server (http://plantgrn.noble.org/psRNATarget/). To expand the utility of sequencing data, sRNAs obtained from the same developmental stage were pooled and were further assembled into a non-redundant set.

### Differential expression analysis of miRNAs

The miRNA expression levels were calculated by TPM (transcript per million) using a normalization formula Normalized expression = Mapped read count/Total reads × 1,000,000)^40^. Differential expression analysis between two samples was carried out with the DESeq R package (1.8.3). Benjamini & Hochberg method was used to adjust the P-values, and 0.05 was set as the threshold for significantly differential expression by default^29^.

### Target prediction, GO enrichment and KEGG pathway analysis

Novel miRNA, significantly expressed and conserved, were selected and used for target prediction using the psRNA Target server by psRobot_tar in psRobot^41^. Target gene candidates of differentially expressed miRNAs were performed through Gene Ontology (GO) enrichment analysis implemented with GOseq based Wallenius non-central hyper-geometric distribution^42^. Moreover, GO terms were also submitted to enrichment analysis by agriGO tool^43^. Functional annotation of miRNA was also performed through KEGG (Kyoto Encyclopedia of Genes and Genomes) pathway analysis. KOBAS software was used to test the statistical enrichment of target gene candidates involved in KEGG pathways^44^.

### Validation of miRNA expression with qRT-PCR analysis

Total RNA was isolated from *R. pulchrum* flowers with TRIzol reagent (Invitrogen) according to the manufacturer’s instructions. The quality and quantity of RNA were tested with agarose gel electrophoresis and ultraviolet spectrophotometer. Mir-X miRNA First-Strand Synthesis Kit and Mir-X miRNA qRT-PCR TB Green Kit were selected (Takara) in this research. Nine miRNAs were randomly selected for real-time qPCR analysis on a Step OnePlus Real-Time PCR system (Applied Biosystems, USA). The 5′ primer were miRNA-specific (Table S8), and the 3′ primer is the mRQ 3′ primer supplied with the kit. Relative expression level of each miRNA was normalized against EF1 expression levels, and fold-change was calculated according to the 2^–ΔΔCT^ method. In particular, three technical replicates were carried out. Real-time PCR reaction was conducted at 95 °C for 5 min, followed by 40 cycles (incubations at 95 °C for 15 s, and 60 °C for 31 s). The dissociation curve was analyzed at 55 °C–95 °C.

### Supplementary Information

Supplementary Figure S1.

Supplementary Figure S2.

Supplementary Figure S3.

Supplementary Figure S4.

Supplementary Figure S5.

Supplementary Figure S6.

Supplementary Legends.

Supplementary Table S1.

Supplementary Table S2.

Supplementary Table S3.

Supplementary Table S4.

Supplementary Table S5.

Supplementary Table S6.

Supplementary Table S7.

Supplementary Table S8.

## Data Availability

The datasets generated and analysed during current study are available in the NCBI database under the BioProject, Biosample, and SRA numbers of PRJNA485857, SAMN09829198-SAMN09829201, and SRR7698534-SRR7698537, respectively.
