# Supplementary figures and images for: Small RNA sequencing provides insights into molecular mechanism of flower development in Rhododendron pulchrum Sweet
Source: Sci Rep. 2023 Oct 20;13:17912. doi: 10.1038/s41598-023-44779-z (PMC10589353; doi:10.1038/s41598-023-44779-z)

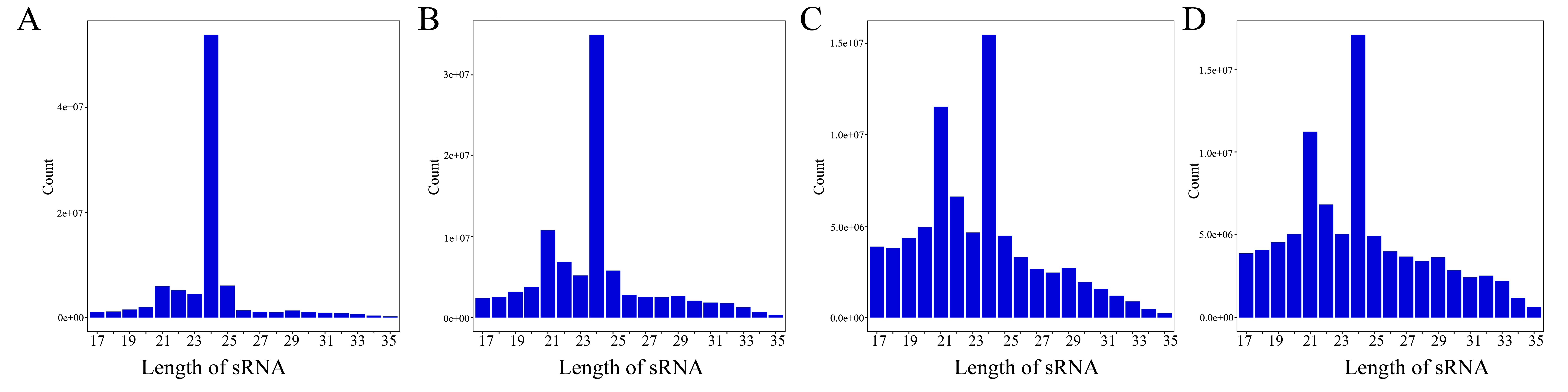

Supplement: Supplementary file 1 — Supplementary Figure S1. [file 41598_2023_44779_MOESM1_ESM.tif]

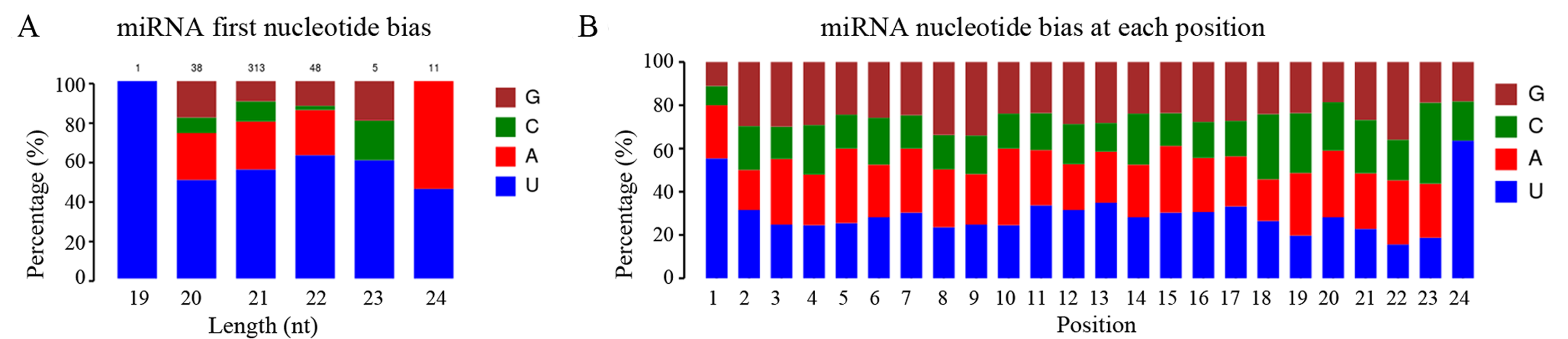

Supplement: Supplementary file 2 — Supplementary Figure S2. [file 41598_2023_44779_MOESM2_ESM.tif]

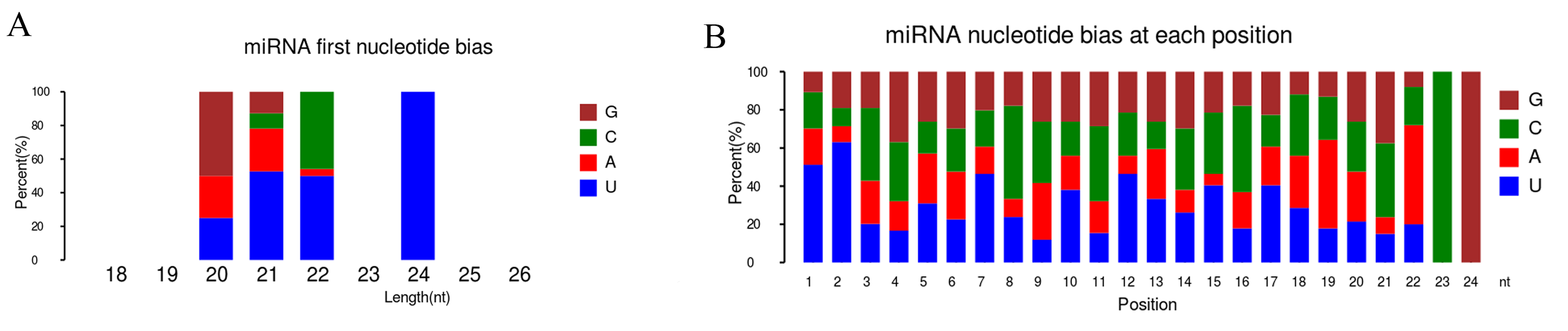

Supplement: Supplementary file 3 — Supplementary Figure S3. [file 41598_2023_44779_MOESM3_ESM.tif]

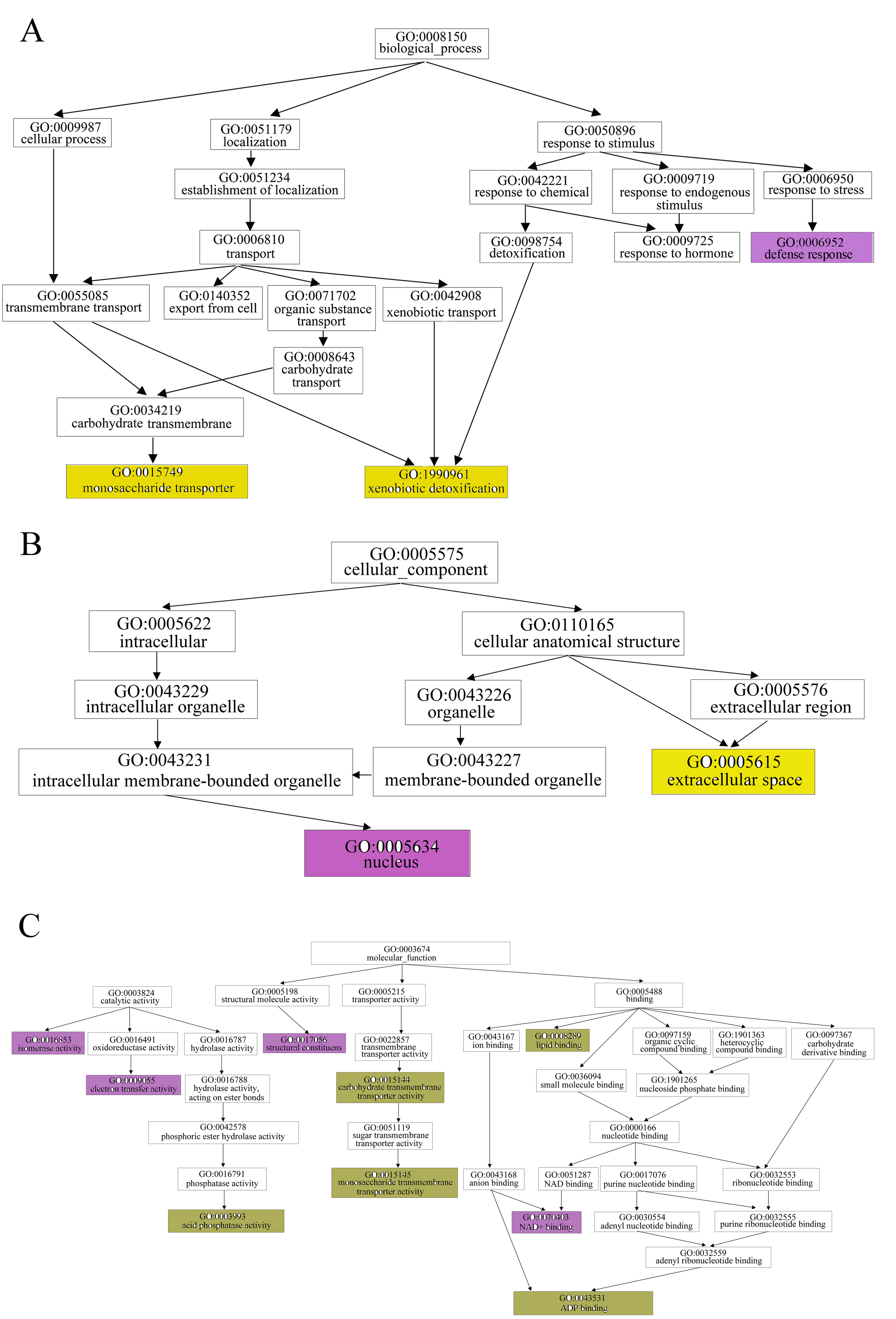

Supplement: Supplementary file 4 — Supplementary Figure S4. [file 41598_2023_44779_MOESM4_ESM.tif]

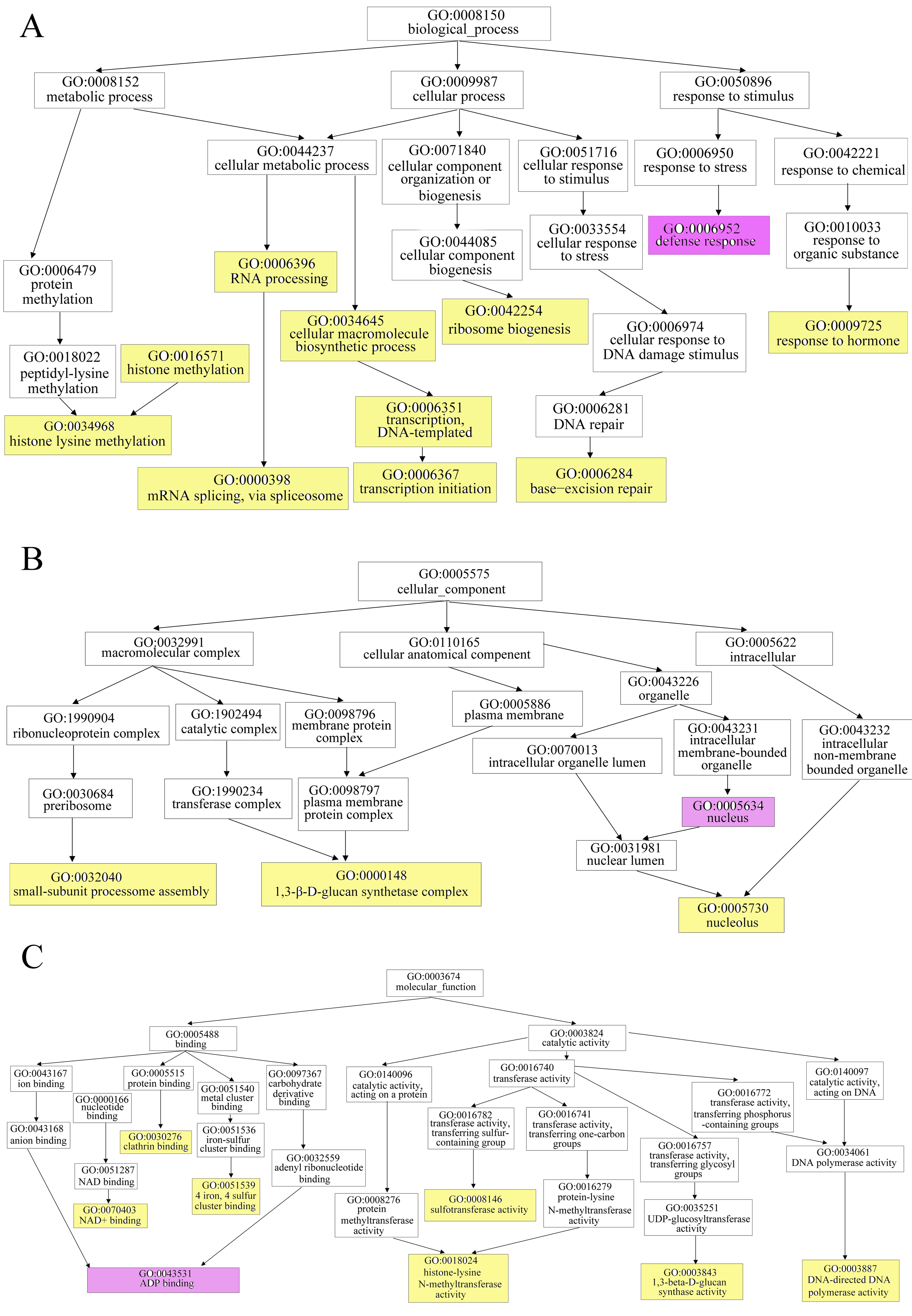

Supplement: Supplementary file 5 — Supplementary Figure S5. [file 41598_2023_44779_MOESM5_ESM.tif]

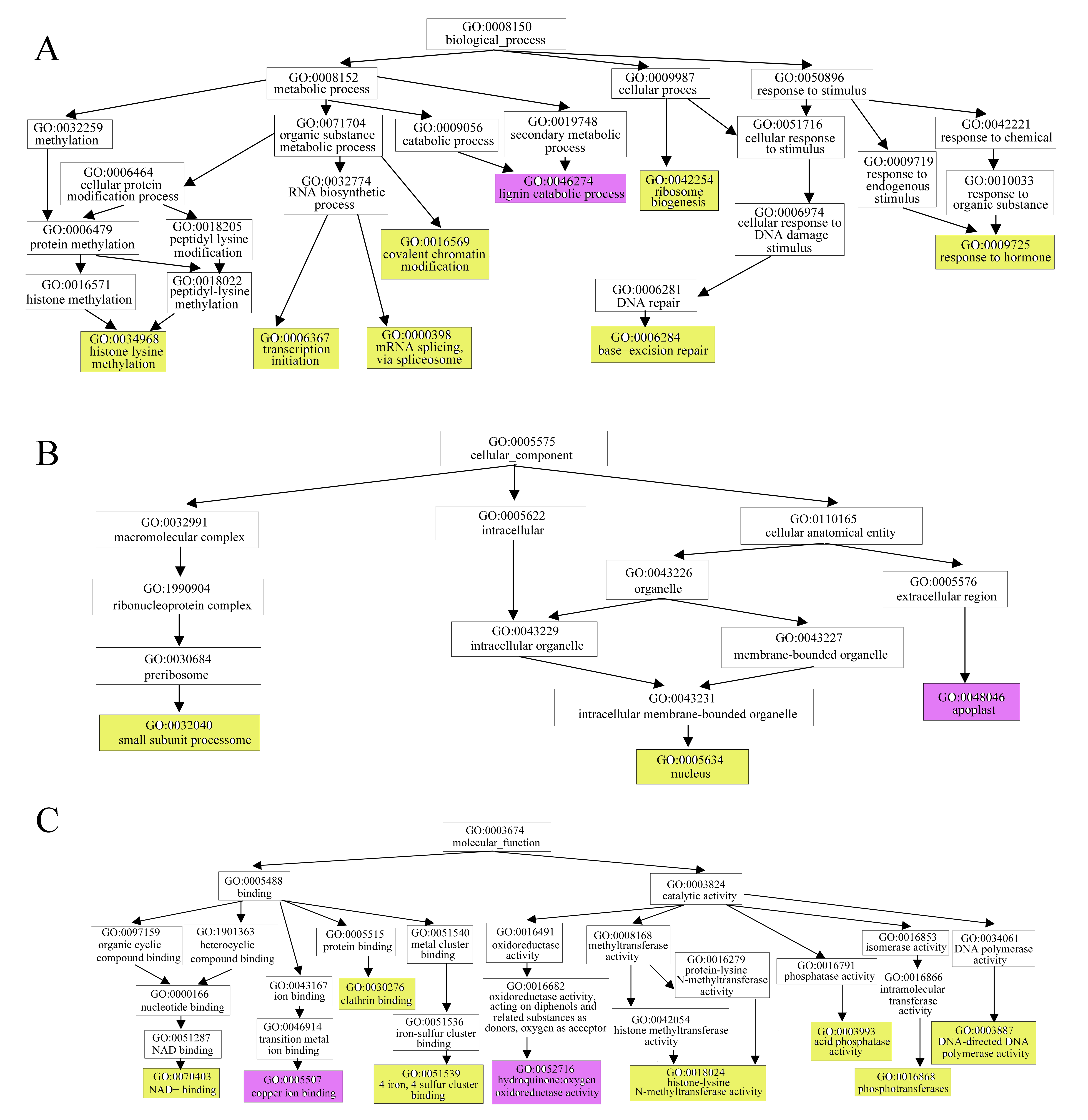

Supplement: Supplementary file 6 — Supplementary Figure S6. [file 41598_2023_44779_MOESM6_ESM.tif]
