# Supplementary material for: Small RNA sequencing provides insights into molecular mechanism of flower development in Rhododendron pulchrum Sweet
Source: Sci Rep. 2023 Oct 20;13:17912. doi: 10.1038/s41598-023-44779-z (PMC10589353; doi:10.1038/s41598-023-44779-z)
Supplement: Supplementary file 7 — Supplementary Legends. [file 41598_2023_44779_MOESM7_ESM.docx]

Figure S1 Length distribution of sRNA in four flower samples at stage I (A), stage II (B), stage III (C), and stage IV (D), respectively.

Figure S2 First nucleotide bias (A) and nucleotide bias at each position (B) of identified miRNA in *R. pulchrum*.

Figure S3 First nucleotide bias (A) and nucleotide bias at each position (B) of novel predicted miRNA in *R. pulchrum*.

Figure S4 GO enrichment analysis of potential miRNA targets of differently expressed miRNAs during *R. pulchrum* flower development from stage I to stage II.

Figure S5 GO enrichment analysis of potential miRNA targets of differently expressed miRNAs during *R. pulchrum* flower development from stage II to stage III.

Figure S6 GO enrichment analysis of potential miRNA targets of differently expressed miRNAs during *R. pulchrum* flower development from stage III to stage IV.
