# Supplementary material for: Small RNA sequencing provides insights into molecular mechanism of flower development in Rhododendron pulchrum Sweet
Source: Sci Rep. 2023 Oct 20;13:17912. doi: 10.1038/s41598-023-44779-z (PMC10589353; doi:10.1038/s41598-023-44779-z)
Supplement: Supplementary file 15 — Supplementary Table S8. [file 41598_2023_44779_MOESM15_ESM.doc]

Table S8 Information on primers of miRNA for real-time qPCR amplification.

| No. | miRNA ID | 5’ Primers (5’-3’) |
| --- | --- | --- |
| 1 | rsi-MIR158-3 | TCCCAAATGTAGACAAAGC |
| 2 | rsi-MIR159-8 | TTTGGATTGAAGGGAGCTCTC |
| 3 | rsi-MIR162-2 | TGGACGCAGCGGTTCATCGATC |
| 4 | rsi-MIR167_1-3 | TGAAGCTGCCAGCATGATCTAA |
| 5 | rsi-MIR171_1-11 | TTGAGCCGTGCCAATATCACT |
| 6 | rsi-MIR396-1 | TTCAAGAAAGCTGTGGGAAG |
| 7 | rsi-MIR398-6 | TGTGTTCTCAGGTCACCCCTT |
| 8 | rsi-MIR535-2 | TGACGACGAGAGAGAGCACGC |
| 9 | rsi-undef-11 | TGCCTGGCTCCCTGTATGCCA |
